# First morphological and molecular identification of third-stage larvae of *Anisakis typica* (Nematoda: Anisakidae) from marine fishes in Vietnamese water

**DOI:** 10.21307/jofnem-2021-010

**Published:** 2021-02-19

**Authors:** Hoang Van Hien, Bui Thi Dung, Ha Duy Ngo, Pham Ngoc Doanh

**Affiliations:** 1 Institute of Ecology and Biological Resources, Vietnam Academy of Science and Technology, Hanoi, Vietnam; 2 Graduate University of Science and Technology, Vietnam Academy of Science and Technology, Hanoi, Vietnam

**Keywords:** Anisakid larvae, Intermediate fish host, Molecular analyzes, Morphology, Vietnam

## Abstract

Anisakid nematodes are parasites of cetaceans, their larval stages live in marine fishes. The third-stage larvae of some *Anisakis* species are also the etiological agents of human anisakiasis caused by consumption of raw or undercooked infected fish. Thus, identiﬁcation of *Anisakis* larvae at the species level is crucial for their ecology and epidemiology. In Vietnam, although *Anisakis* larvae have been reported, they have not been identified to the species level. The aim of this study was, therefore, to identify third-stage larvae of *Anisakis* collected from marine fishes in Vietnamese water, based on morphological characteristics and molecular analysis. All *Anisakis* larvae found in this study were morphologically similar to each other and identical to *Anisakis typica*. In addition, molecular analysis based on ITS1-5.8S-ITS2 sequences confirmed them as *A. typica*. Vietnamese *A. typica* population was genetically close to those from Asian countries and Australia. The third-stage larvae of *A. typica* were collected from eight fish species from three localities in the South of Vietnam. Among them, seven were recorded as new intermediate hosts of *A. typica*. This is the first identification of *A. typica* larvae in Vietnamese water with records of new fish hosts.

Nematodes of the genus *Anisakis* (Nematoda: Anisakidae) are parasites of marine organisms. The life cycle of these nematodes requires marine mammals, mainly cetaceans, as the definitive hosts, and crustaceans, ﬁsh, and cephalopods as intermediate/paratenic hosts (Klimpel and Palm, 2011). Humans are accidental hosts due to ingestion of raw or undercooked ﬁsh containing the third infective-stage larvae (L3). Human anisakiasis patients suffer from abdominal pain, nausea, vomiting, and/or diarrhea (Dorny et al., 2009). In addition, allergic reactions may occur due to exposure to the nematode antigens (Aibinu et al., 2019; Audicana et al., 2002). Given the influence on human health, *Anisakis* nematodes are of interest. Anisakid larvae can be morphologically identified at the genus level by typical characteristics of anterior and posterior regions, and are classiﬁed into two types, type I and II, based on the length of the ventriculus and presence/absence of the tail spine (mucron): *Anisakis* type I larva has a longer ventriculus and a mucron, while type II larva has a shorter ventriculus and no mucron (Berland, 1961). Type I consists of *A. simplex*, *A. pegreffii*, *A. typica*, *A. ziphidarum*, and *A. nascettii*, while type II consists of *A. paggiae*, *A. physeteris*, and *A. brevispiculata* (Mattiucci and Nascetti, 2008). Previously, it was not easy to identify anisakid larvae at the species level, because there is a lack of distinct morphological characteristics required for species identification (Farjallah et al., 2008). Recent studies indicated morphological differences between *Anisakis* species (Chen and Shih, 2015; Sonko et al., 2019; Tunya et al., 2020). In addition, molecular tools allow the accurate identification of anisakid larvae by using sequences of the internal transcribed spacer (ITS) region of ribosomal DNA (D’amelio et al., 2010; Mattiucci and Nascetti, 2008).

In Vietnam, data on *Anisakis* nematodes are scarce. There have been a few reports on *Anisakis* larvae without morphological description and identification to species level (Arthur and Te, 2006; Ngo et al., 2009). During our recent comprehensive survey for parasites of marine fishes in Vietnamese water, we collected *Anisakis* larvae from eight fish species. The aim of the present study was to identify these *Anisakis* specimens from Vietnamese water by morphological and molecular approaches.

## Materials and methods

### Fish examination and larval collection

**Figure 1:**
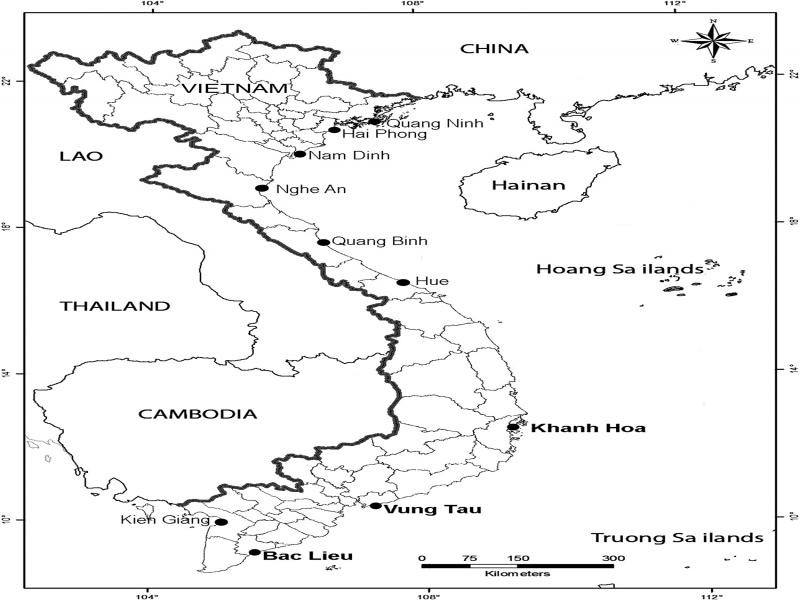
Study sites along the seashore of Vietnam. Three localities where fishes were infected with *Anisakis* larvae are print in bold.

Marine fish were bought in 10 fish ports located in 10 provinces along the seashore of Vietnam where fishing vessels docked (Fig. 1). All fish specimens were placed on ice and transferred to the laboratory under good aeration. Fish were dissected, their body cavities and internal organs were examined under a stereomicroscope. Third-stage larvae were isolated from the body cavity and visceral organs. The larvae were washed in phosphate-buffered saline. For morphological identiﬁcation, larvae were preserved in 4% formalin. Representative specimens were preserved in 70% ethanol for DNA isolation.

### Morphological study

*Anisakis* larvae were soaked in a solution of glycerin-phenol-lactic acid-distilled water (2:1:1:1) for about 48 hr until the body parts were transparent. Then, the larvae were observed and measured under a light microscope (ECLIPSE H600 L Nikon). For scanning electron microscopy, *Anisakis* larvae were prepared according to Madden and Tromba (1976) and Morsy et al. (2017). Larvae were identiﬁed according to the reported references (Berland, 1961; Chen and Shih, 2015; Mattiucci et al., 2009; Sonko et al., 2019; Tunya et al., 2020).

### Molecular and phylogenetic analysis

DNA of three representative larvae from three localities was extracted using QIAamp DNA stool Minikit (Qiagen, Hilden, Germany). Two primers NC5-GTAGGTGAACCTGCGGAAGGATCATT (forward) and NC2-TTAGTTTCTTTTCCTCCGCT (reverse) were used in a polymerase chain reaction (PCR) to amplify the rDNA region of the first to the second internal transcribed spacer (ITS1-5.8S-ITS2) (Zhu et al., 2000). PCR products were electrophoresed in a 1.0% agarose gel and visualized by ethidium bromide staining. Positive PCR products were sent to Macrogen Company (Korea) for sequencing. The nucleotide sequences obtained in this study were deposited in GenBank under accession numbers LC592876-LC592878.

BLAST searches were performed at NCBI (http://blast.ncbi.nlm.nih.gov/Blast.cgi) to find sequence similarities. Sequences of *Anisakis* species available in GenBank were downloaded for analysis. The analysis involved 42 nucleotide sequences, including a sequence (KM491173) of *Contracaecum osculatum* as an out-group. The evolutionary history was inferred by using the Maximum Likelihood method based on the Kimura 2-parameter model in MEGA software v.7.0. (Kumar et al., 2016). Initial tree(s) for the heuristic search were obtained automatically by applying Neighbor-Join and BioNJ algorithms to a matrix of pairwise distances estimated using the Maximum Composite Likelihood approach, and then selecting the topology with superior log likelihood value. A discrete Gamma distribution was used to model evolutionary rate differences among sites (five categories (+*G*, parameter = 0.8044)). All positions containing gaps and missing data were eliminated. There were a total of 656 positions in the final dataset.

## Results and discussion

A total of 3,775 fish of 138 species from 10 study sites were examined. *Anisakis* larvae were found from eight fish species from three localities, Khanh Hoa, Vung Tau, and Bac Lieu, in the South of Vietnam. Prevalences of infection ranged from 10 to 50% with intensity varied from 1 to 19 larvae/fish (Table 1), non-infected fish species were presented in the supplemental material.

**Table 1.** Prevalence of *Anisakis* larvae infection in marine fishes in Vietnamese water.

Locality	No. of fish examined	No. of fish species	Infected fish species	No. of infected/examined fish (%)	Density
Quang Ninh	615	62	0		
Hai Phong	478	41	0		
Nam Dinh	122	23	0		
Nghe An	303	50	0		
Quang Binh	520	75	0		
Hue	211	28	0		
Khanh Hoa	766	82	*Dcapterus macarellus*	10/20 (50.0)	1-19
			*Trichiurus lepturus*	6/20 (30.0)	1-5
			*Sargocentron rubrum*	1/10 (10.0)	1
			*Lutjanus johnii*	1/10 (10.0)	1
			*Megalaspis cordyla*	2/12 (16.7)	1; 3
			*Priacanthus hamrur*	1/8 (12.5)	2
			*Pristipomoides filamentosus*	3/10 (30.0)	1; 1; 1
Vung Tau	40	6	*Megalaspis cordyla*	1/5 (20.0)	6
Bac Lieu	390	58	*Carangoides malabaricus*	1/10 (10.0)	1
Kien Giang	330	63	0		
Total	3775	138	8		1-19

**Figure 2:**
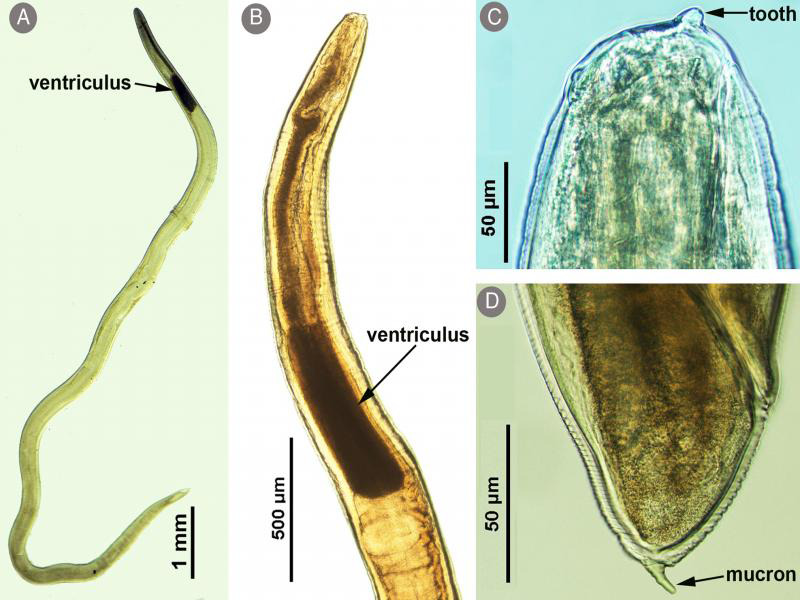
Light micrographs of *Anisakis typica* larva. A. Whole larva; B. Anterior part of the body showing a long ventriculus; C. Anterior part of the body showing a boring tooth; D. Posterior end of the body showing a mucron.

**Figure 3:**
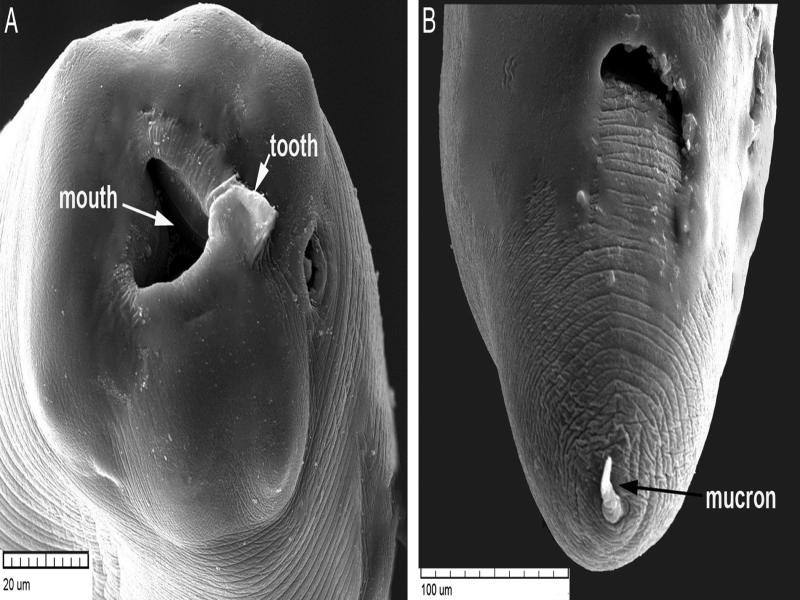
Scanning electron micrographs of *Anisakis typica* larva. A. Anterior end showing a mouth and a boring tooth; B. Posterior end showing a mucron.

All *Anisakis* larvae were morphologically similar to each other. The body of the larvae was cylindrical in shape, attenuated at both ends, and measured 17.2 to 20.3 (18.6 ± 1.1) mm long and 0.26 to 0.34 (0.29 ± 0.03) mm width (*n* = 30 larvae). The lips were inconspicuous, with a prominent boring tooth at the anterior extremity. The esophagus had an anterior muscular part and measured 1.52 to 1.58 (1.54 ± 0.02) mm long and a glandular ventriculus measured 0.58 to 0.82 (0.64 ± 0.04) mm long. Long intestinal caeca with clear demarcation were present. The body of larvae ended at a short cylindrical mucron measuring 0.021 to 0.030 (0.025±0.005) mm long (Figs. 2, 3). These characteristics of the third-stage larvae were identical to *Anisakis* larvae type I (Berland, 1961). It has previously been noted that it is difficult to distinguish between *Anisakis* species belonging to type I because they look quite similar to each other (Farjallah et al., 2008). However, recent studies based on morphological and molecular approaches provided descriptions and microphotographs showing differences between L3 larvae of *A. pegreffii* and *A. typica* (Chen and Shih, 2015; Sonko et al., 2019). In addition, Tunya et al. (2020) suggested that the protruded mucron of L3 larvae can be used to identify anisakid larvae at the species level: the protruded mucron of *A. simplex* was cone-shape, while that of *A. typica* was cylindrical-shape, which is narrower and longer than that of *A. simplex*. According to these, L3 larvae of *Anisakis* specimens found in this study were identified as *A. typica*.

**Figure 4:**
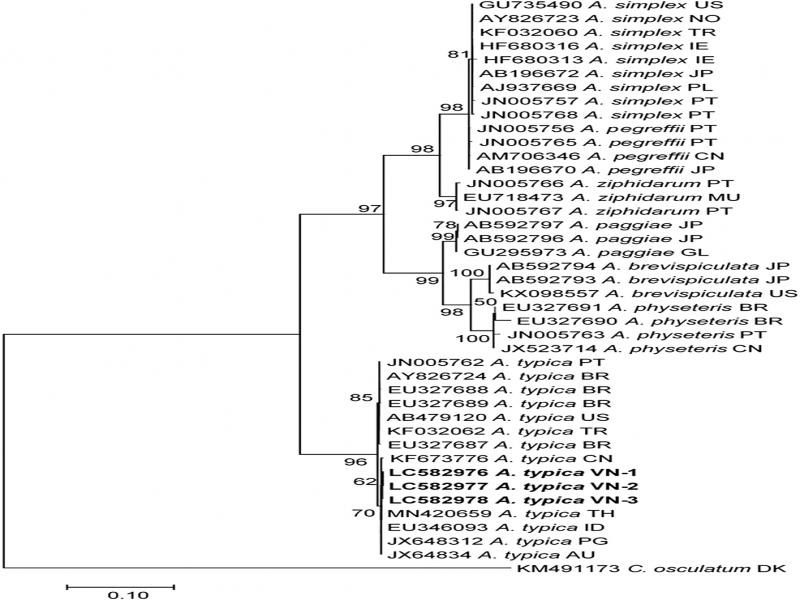
Phylogenetic tree reconstructed from ITS1-5.8S-ITS2 sequences of *Anisakis typica* from Vietnam and other *Anisakis* species. The tree is drawn to scale, with branch lengths measured in the number of substitutions per site. Bootstrap values are shown above the nodes. The nucleotide sequences obtained in this study are printed in bold, and others from the GenBank database are shown with Accession No., species name, and two letter country code of their geographical origin (AU: Australia, BR: Brazil, CN: China, DK: Denmark, GL: Greenland, IN: Indonesia, IE: Ireland, JP: Japan, MU: Mauritius, NO: Norway, PG: Papua New Guinea, PL: Poland, PT: Portugal, TH: Thailand, TR: Turkey, US: United States of America, VN: Vietnam).

Because the *Anisakis* larvae collected in the present study were all morphologically similar to each other, three larvae representative for three locations were used for molecular analyses. Three ITS1-5.8S-ITS2 sequences obtained from three L3 larvae were 771 bp and completely identical (100%) with each other. In agreement with morphological identification, the BLAST searches revealed that the ITS1-5.8S-ITS2 sequences of *Anisakis* larvae from Vietnam showed the highest similarity (100%) with that of *A. typica* available in GenBank. The analysis of genetic distances demonstrated that inter-speciﬁc genetic distances between *A. typica* and other *Anisakis* species were: *A. paggiae* 17.2%; *A. ziphidarum* 17.3%; *A. pegrefﬁi* 18.0%; *A. simplex* 18.0%; *A. physeteris* 18.4%; and *A. brevispiculata* 18.7%. In the phylogenetic tree (Fig. 4), *A. typica* made a distinct clade that was far distant from other *Anisakis* species. Vietnamese *A. typica* were genetically close to those from China, Thailand, Indonesia, Papua New Guinea, and Australia, to make a common group that was separated from another group of America, Brazil, Turkey, and Portugal. Our analysis is in agreement with a previous report that the separation of *A. typica* populations related to geographical origins (Tunya et al., 2020).

Molecular studies of *Anisakis* from various parts of the world Oceans confirmed the validity of nine *Anisakis* species (Klimpel and Palm, 2011). Most of them distribute in the Atlantic and the Mediterranean Sea with several records of some species in the South American, African, and Australian sea, and SW Paciﬁc Ocean. A unique distribution pattern has been known for *A. typica* which has been reported in warmer temperate and tropical waters (Mattiucci and Nascetti, 2006). In Asian countries, *A. typica* larvae have been reported in Japan, Korea, China, Taiwan, Indonesia, and Thailand (Lee et al., 2016; Palm et al., 2008, 2017; Sonko et al., 2019; Tunya et al., 2020; Umehara et al., 2010; Zhu et al., 2007). In the present study, we firstly identified *A. typica* larvae in Vietnamese water. Although a limited number of larval samples were molecularly analyzed, we speculated, based on their morphological similarity, that the *Anisakis* larvae found in this study are all *A. typica*. It is highly possible that *A. typica* is the most dominant species or the only *Anisakis* species in the South of Vietnamese water similar to reports in Thailand water where *A. typica* was the only species found in the Gulf of Thailand (Eamsobhana et al., 2018; Tunya et al., 2020).

Regarding intermediate fish hosts, the third-stage larvae of *A. typica* are found in various fish species. They differ from place to place depending on geographical locations. Outside of Vietnamese water, 32 fish species have been reported as intermediate hosts of *A. typica* (Table 2). In this study in Vietnam, *A. typica* larvae were found from eight fish species, *Carangoides malabaricus*, *Dcapterus macarellus*, *Sargocentron rubrum*, *Lutjanus johnii*, *Megalaspis cordyla*, *Priacanthus hamrur*, *Pristipomoides filamentosus*, and *Trichiurus lepturus.* Among these, only *T. lepturus* has been previously reported as an intermediate host of *A. typica* in Korean and Taiwanese waters, the other seven species are reported as new hosts.

**Table 2.** Intermediate fish hosts of *Anisakis typica* in the World and in Vietnam.

No.	Host species	Localities	References
1	*Sotalia guianensis*		
2	*Auxis thazard*		
3	*Thunnus thynnus*	Brazil Coast	
4	*Pseudopercis numida*		
5	*Trachurus picturatus*		
6	*Scomber japonicus*	Portugal	Mattiucci et al. (2002), Marques et al. (2006), Pantoja et al. (2015)
7	*Platichthys flesus*		
8	*Scomberomorus commerson*		
9	*Euthynnus affinis*		
10	*Sarda orientalis*	Somalia	
11	*Coryphaena hippurus*		
12	*Stenella attenuata*		
13	*Globicephala macrorhynchus*	Florida	Mattiucci et al. (2005)
14	*Scomber scombrus*		
15	*Merluccius merluccius*	North Africa	Farjallah et al. (2008)
16	*Phycis phycis*		
	*Scomber japonicus*	Turkey	Pekmezci et al. (2014)
17	*Micromesistius poutassou*		
	*Trichiurus* spp.	Japan	Umehara et al. (2010)
	*Scomber japonicus*		Suzuki et al. (2010)
18	*Trichiurus lepturus*	Korea	Lee et al. (2009)
19	*Todarodes pacificus*		
20	*Astroconger myriaster*		Cho et al. (2015)
21	*Decapterus macarellus*		
22	*Gerres oblongus*		
23	*Pinjalo lewisi*		
24	*Pinjalo pinjalo*	Papua New Guinea	Koinari et al. (2013)
25	*Selar crumenophthalmus*		
26	*Scomberomorus maculatus*		
27	*Thunnus albacares*		
28	*Auxis rochei rochei*	Indonexia	Palm et al. (2008)
29	*Decapterus russelli*		
30	*Nemipterus hexodon*	Thailand	Tunya et al. (2020)
31	*Nemipterus japonicus*		
32	*Scomber australasicus*	Taiwan	Umehara et al. (2010), Sonko et al. (2019)
	*Trichiurus lepturus*		
1	*Carangoides malabaricus*		
2	*Dcapterus macarellus*		
3	*Sargocentron rubrum*		
4	*Lutjanus johnii*	Vietnam	In this study
5	*Megalaspis cordyla*		
6	*Priacanthus hamrur*		
7	*Pristipomoides filamentosus*		
	*Trichiurus lepturus*		

## Conclusion

The present study firstly identified *A. typica* larvae, based on morphological characteristics and molecular analysis, from eight marine fish species in the South of Vietnamese water and recorded seven fish species as new intermediate hosts of this *Anisakis* nematode. Genetically, the ITS1-5.8S-ITS2 sequences of Vietnamese *A. typica* were close to those from Asian countries and Australia, to make a common group separated from another group from America and Europe.
